# Mechanics Based Tomography: A Preliminary Feasibility Study

**DOI:** 10.3390/s17051075

**Published:** 2017-05-09

**Authors:** Yue Mei, Sicheng Wang, Xin Shen, Stephen Rabke, Sevan Goenezen

**Affiliations:** 1 Department of Mechanical Engineering, Texas A&M University, College Station, TX 77843, USA; meiyue1989@gmail.com (Y.M.); sx1992@tamu.edu (X.S.); pokeg16@tamu.edu (S.R.); 2 Department of Mathematics, Texas A&M University, College Station, TX 77843, USA; sichengwang0223@gmail.com

**Keywords:** digital image correlation systems, DIC, force sensors, boundary displacement tracking, non-destructive material characterization, inverse problems, parameter identification, incomplete data

## Abstract

We present a non-destructive approach to sense inclusion objects embedded in a solid medium remotely from force sensors applied to the medium and boundary displacements that could be measured via a digital image correlation system using a set of cameras. We provide a rationale and strategy to uniquely identify the heterogeneous sample composition based on stiffness (here, shear modulus) maps. The feasibility of this inversion scheme is tested with simulated experiments that could have clinical relevance in diagnostic imaging (e.g., tumor detection) or could be applied to engineering materials. No assumptions are made on the shape or stiffness quantity of the inclusions. We observe that the novel inversion method using solely boundary displacements and force measurements performs well in recovering the heterogeneous material/tissue composition that consists of one and two stiff inclusions embedded in a softer background material. Furthermore, the target shear modulus value for the stiffer inclusion region is underestimated and the inclusion size is overestimated when incomplete boundary displacements on some part of the boundary are utilized. For displacements measured on the entire boundary, the shear modulus reconstruction improves significantly. Additionally, we observe that with increasing number of displacement data sets utilized in solving the inverse problem, the quality of the mapped shear moduli improves. We also analyze the sensitivity of the shear modulus maps on the noise level varied between 0.1% and 5% white Gaussian noise in the boundary displacements, force and corresponding displacement indentation. Finally, a sensitivity analysis of the recovered shear moduli to the depth, stiffness and the shape of the stiff inclusion is performed. We conclude that this approach has potential as a novel imaging modality and refer to it as Mechanics Based Tomography (MBT).

## 1. Introduction

Medical imaging modalities map the tissue’s interior to visualize tissue composition and detect diseased tissues based on image contrasts. Computed tomography and X-rays rely on changes in tissue density or chemical composition. Magnetic resonance imaging provides image contrasts based on the tissue’s water concentration that may vary between tissue types and within diseased tissue regions [1]. In ultrasound imaging, acoustic waves are transmitted and reflected at tissue interfaces and microstructural constituents. Time of flight of reflected signals are computed and B-mode images constructed from acoustic pressure wave intensities.

A more recent medical imaging modality is based on tissue stiffness by mapping the Young’s modulus, Poisson’s ratio, or other mechanical properties spatially from known displacement fields [2,3,4,5,6,7,8,9,10,11,12], widely termed as elastography. These maps may provide additional and complementary information to classify tissue types or detect diseased tissues based on changes in their material property distribution. The displacement field can be computed from imaging modalities such as ultrasound, optical coherence tomography, or magnetic resonance imaging [13,14,15,16,17,18]. The displacement field can be determined in the entire imaging domain, and data can be acquired in two-dimensional space or three-dimensional space if the imaging device is three-dimensional, or from a stack of two-dimensional data. One stream of elastography focuses on strain imaging, where the gradient of the displacement field is computed and correlated directly to the reciprocal of the stiffness distribution qualitatively without rigorously considering the laws of physics describing the solid. The other stream of elastography focuses on solving inverse problems using physics-based equations, e.g., equations of equilibrium or motion [3,4,9,11,19,20,21].

The authors of this paper have recently developed a novel approach to “sense” the stiffness distribution of tissues using measured force indentations, boundary displacements from digital camera systems, and mechanics-based mathematical algorithms [22,23]. Three-dimensional displacements on the specimen’s boundary (i.e., surface) can be determined with two or more digital cameras with images taken before and after applying a force to deform the tissue region, implying that three dimensional stiffness distribution can be mapped without the need of stacking two dimensional image slices. Displacement acquisition with digital cameras is widely known as digital image correlation (DIC) systems and provides highly precise boundary displacements or strains [24,25] since images from currently available regular cameras have high resolutions. DIC based research often focuses on strains on the specimen’s boundary and is utilized to detect changes in material properties. This approach has strong limitations, since strains do not represent the actual material properties of the solid and depend on the geometry and boundary conditions. Furthermore, the strain in the interior of the specimen is not known, thus further limits the capability of using the strain as a qualitative measure of stiffness.

In our novel approach, the stiffness values and, in particular, the shear modulus values, were assumed to be unknown at the nodes and interpolated with finite element shape functions throughout the entire problem domain. Thus, the total number of unknown shear modulus values was equal to the total number of finite element mesh nodes, resulting in a very large number of unknowns in total. While from a purely mechanical point of view, it would be expected that such an approach would yield non-unique solutions, a successful reconstruction was possible by utilizing multiple boundary displacement data sets from multiple indentations, sequentially applied at distinct locations around the specimen. Thus, we term this approach as Mechanics Based Tomography (MBT). The boundary displacements were assumed to be known everywhere on the specimen’s boundary, and it was feasible to recover an inclusion embedded in a homogeneous background. A thorough literature review for related work was provided in our previous publication for the interested reader [22], and, to the best of our knowledge, no progress has been reported in the literature related to this work.

We recognize that it is not convenient to determine displacements everywhere on the boundary since it requires several cameras to image the entire boundary of the specimen. Furthermore, the deformations were induced by applying a displacement indentation, and no force or traction was assumed to be known, resulting in a shear modulus reconstruction being off by a multiplicative factor [26]. Furthermore, only one inclusion in a homogeneous background was simulated. Even though we did not assume any particular form of inhomogeneity, the question may still arise if this approach has the capability to recover more than one inclusion.

In this paper, we will test the feasibility to recover the shear modulus distribution (1) of one or two inclusions; (2) absolutely (i.e., quantitatively) by including known (measured) force indentations; (3) utilizing boundary displacements from partial boundaries of the specimen for convenient data collection; and (4) utilizing a curved boundary domain. The paper is organized as follows: in Section 2, we introduce the mathematical foundation along with the computational procedure for the inverse algorithms; in Section 3, we test the inverse algorithms with simulated experiments for various geometric domains and shear modulus distributions; in Section 4, we discuss the numerical results and end with conclusions in Section 5.

## 2. Inverse Algorithms Using Limited Boundary Displacements

In this paper, the material is assumed to be isotropic, heterogeneous, linear elastic, and in the state of incompressible plane strain. Thus, the forward problem is posed to find the displacements and hydrostatic pressure such that the equilibrium equations are satisfied. For a discretized problem domain, we use finite element methods to solve the forward problem in elasticity, resulting in the following linear algebraic equations:

(1) KU=f

where **K** and **f** are the global stiffness matrix and the force vector, respectively. **U** is the nodal unknown vector including the displacement components in both directions together with the pressure. To address volumetric locking induced by incompressibility, the global stiffness matrix will be augmented with terms arising from a residual term referred to as stabilization according to [27]. This allows us to use the same order interpolation functions to approximate the displacement and pressure variables.

The inverse problem is posed as a constrained optimization problem where the equilibrium equations represent the constraints of the problem. This requires measurements of displacements, acquired here merely on the boundary. Furthermore, applied forces to induce boundary displacements are assumed to be known as well. The objective function to be minimized is given in discretized form using finite element based approximation techniques as follows:

(2) $$F=\sum_{i=1}^{n}\left(\sum_{e=1}^{\bar{N}}w_{e}^{i}{\left(\Delta u_{e}^{i}\right)}^{2}\;\right)+\alpha \sum_{e=1}^{N_{n}}\int_{\Omega_{e}}\sqrt{\sum_{j=1}^{{\bar{N}}_{e}}|\mu_{j}^{e}\nabla N_{j}^{e}(x){|}^{2}+c^{2}}\;d\Omega$$

where *n*, $\bar{N}$, ${\bar{N}}_{e}$ and $N_{n}$ are the total number of data sets (corresponding to the total number of experiments) , the total number of nodes on the measured boundary, the number of element nodes in the domain, and the total number of elements throughout the problem domain, respectively. $\Delta u_{e}^{i}=u_{e}^{i}-{\left(u_{e}^{i}\right)}_{meas}$ is the misfit between the computed displacement $u_{e}^{i}$ and the measured boundary displacement ${\left(u_{e}^{i}\right)}_{meas}$ for the *i*-th simulated experiment. Furthermore, $w_{e}^{i}$ denotes a weight that arises from the finite element interpolation as well as the local element coordinates discussed in detail in [22]. To ensure uniqueness of the final solution of the inverse problem, it is recommended to have a large value of *n* in Equation (2). We note that each experiment may result in displacement measurements at different boundaries, and thus $\bar{N}$ may change for each *i*.

The second term on the right-hand side represents the total variation diminishing regularization term and is a function of the nodal shear modulus $\mu_{j}^{e}$, and c=0.01 is a small constant to ensure that the regularization term is differentiable. Note that the shear moduli are nodal unknowns and interpolated with the same shape functions $N_{j}^{e}(x)$ used for the displacement and pressure variables. The reason for the selection of total variation diminishing regularization is that this regularization type is capable of preserving sharp stiffness contrasts and worked well for us in past studies. This term penalizes the final solution and controls the smoothness of the reconstruction results by choosing a proper regularization factor α. If the regularization factor is chosen to be too large, the final solution will be over-smoothed and the approximated material properties will significantly underestimate the target distribution. On the contrary, if the regularization factor is too small, the final solution will strongly oscillate, since computed displacements are then correlating with noisy measurements in the first term of Equation (2). Approaches such as the L-curve method [28,29], Morozov’s discrepancy principle [30,31] or a smoothness criteria [4,11] provide guidelines to select an optimal regularization factor. In this paper, the regularization factor is chosen based on the smoothness criteria, and the appropriate regularization factor is estimated such that a small sub-region neither oscillates nor overpenalizes the shear modulus distribution. More specifically, at the beginning, we will adopt a large regularization factor and this factor will lead to over smooth results. We will then incrementally decrease the factor. The final regularization factor is selected in a neighborhood of regularization factors, with values below the selected one leading to visible oscillations, while values above this selection lead to no significant changes in smoothness.

To solve the optimization problem, the limited BFGS (Broyden–Fletcher–Goldfarb–Shanno) method is utilized [32,33], which requires the evaluation of the objective function and the nodal gradient of the objective function with respect to the shear modulus. For a discretized problem domain, the straightforward calculation of the gradient is computationally intensive and requires solving the forward problem for each nodal shear modulus at every minimization call. Thus, the adjoint method is utilized, which dramatically reduces the computational cost [4,34,35]. The adjoint method has been thoroughly discussed and universally applied to solve linear and nonlinear inverse problems, and thus will not be further discussed in this paper. The limited BFGS method will update the elastic property distribution and the process is repeated until the functional drops or the gradient is smaller than a defined tolerance.

In the present work, we will mainly focus on testing the feasibility of these inverse algorithms to recover the shear modulus distribution of problem domains having one or two inclusions. To this end, indentation experiments will be simulated by solving the forward problem in elasticity and boundary displacements will be extracted and used as measured displacements. The forward problem is solved using standard finite element methods, with the assumption that the material is incompressible and in a state of plain strain. Comparing this work to our previous publications [10,23], we also assume that the applied force is known together with the induced displacement at that point. As such, the resulting shear modulus distribution will be recovered quantitatively. Finally, we add the same noise level to the simulated boundary displacements (random noise), force and corresponding displacement indentation to study the sensitivity of the mapped shear moduli to noisy data.

The inverse problem is solved using in-house written FORTRAN programs with integrated open source limited BFGS algorithms on shared memory using OpenMP. The total time to solve the inverse problem depends on the total number of boundary displacement datasets and the total number of cores used. For one boundary displacement dataset, it approximately takes an hour to converge fully to the final solution.

## 3. Numerical Results with Simulated Experiments

### 3.1. Case 1: A Square Model with a Small Inclusion

**Figure 1:**
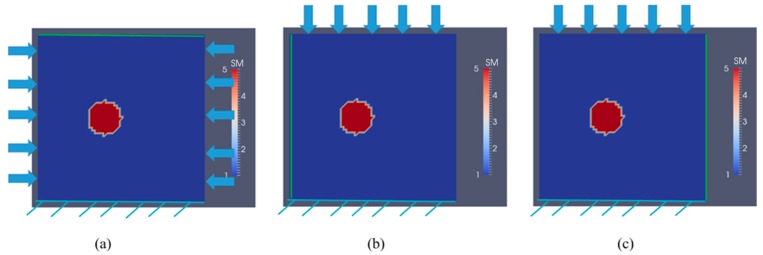
The problem domain with a stiff inclusion surrounded by a soft background. The arrows indicate the indentation locations, and the green line represents the side of known or measured displacements. (**a**) the indentations are sequentially applied pairwise at both lateral sides (net force is zero), and we utilize boundary displacements on the top edge as measured data; (**b**) the indentation is applied on the top edge, and we utilize boundary displacements on the left edge as measured data; (**c**) the indentation is applied on the top edge, and we utilize boundary displacements on the right edge as measured data (unit in the scale bar: 10 kPa). Note: “SM” stands for shear modulus.

First, we consider a 1 cm × 1 cm square with a small inclusion with a radius of 0.1 cm surrounded by a softer homogeneous background material as shown in Figure 1. The coordinate of the center of the inclusion is (0.4 cm, 0.5 cm), the target shear modulus value of the background is 10 kPa and the stiffness in the inclusion is 50 kPa. With regards to boundary conditions, we fix the bottom edge in both directions for all simulations. In Figure 1a, forces are applied pairwise on the left and right side simultaneously and are aligned horizontally but in the opposite direction (net force is zero). Each pairwise applied force induces a displacement on the top boundary (see the green line in Figure 1a). Varying the location of the pairwise applied forces vertically and sequentially provides a rich number of boundary displacement data sets on the top face (green line). In Figure 1b, single force indentations are applied on the top boundary edge sequentially, in order to induce boundary displacements (for each single force indentation) on the left boundary edge (see green vertical line in Figure 1b). In Figure 1c, single force indentations are applied on the top boundary edge sequentially, in order to induce boundary displacements (for each single force indentation) on the right boundary edge (see green vertical line in Figure 1c. Varying the location of applied force indentation as shown in Figure 1b,c, we obtain a rich boundary displacement data set. Simulated displacement measurements are highlighted on the boundary edge with a green line as shown in Figure 1a–c. Furthermore, each indentation induces a force of 0.05 N on the corresponding node in the problem domain. This force will induce small deformations that are suitable for displacement measurements using a digital image correlation system. The problem domain is discretized with 7200 linear triangular elements (61 nodes are uniformly distributed in each direction). The boundary displacement is assumed to be measured on the edge with no applied force indentation. In standard indentation tests, the displacement at the indentation can be measured with high accuracy; therefore, this information will also be included in the inverse solution process.

**Figure 2:**
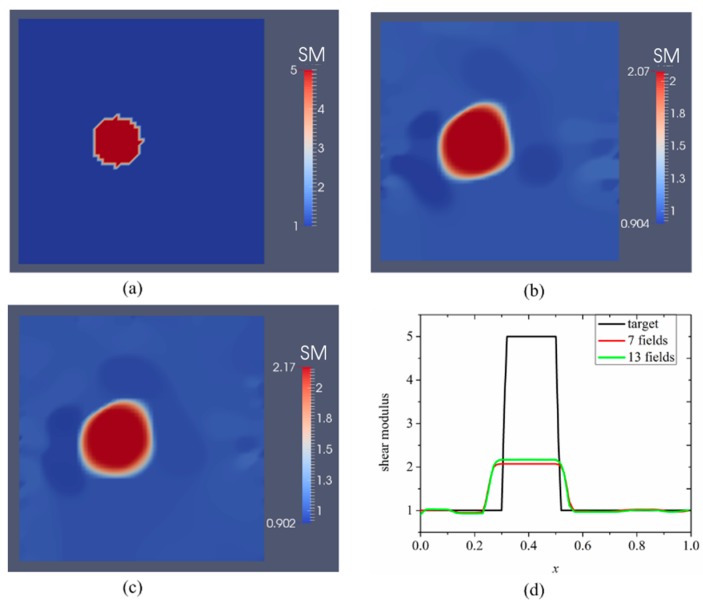
Shear modulus reconstructions for 0.1% noise. (**a**) target shear modulus distribution for comparison; (**b**,**c**) reconstructed shear modulus distribution using 7 and 13 boundary displacement data sets, respectively; (**d**) shear modulus plot over the horizontal line through the center of the inclusion for the target and reconstructed shear modulus distribution (unit in the scale bar: 10 kPa). Note: “SM” stands for shear modulus.

**Figure 3:**
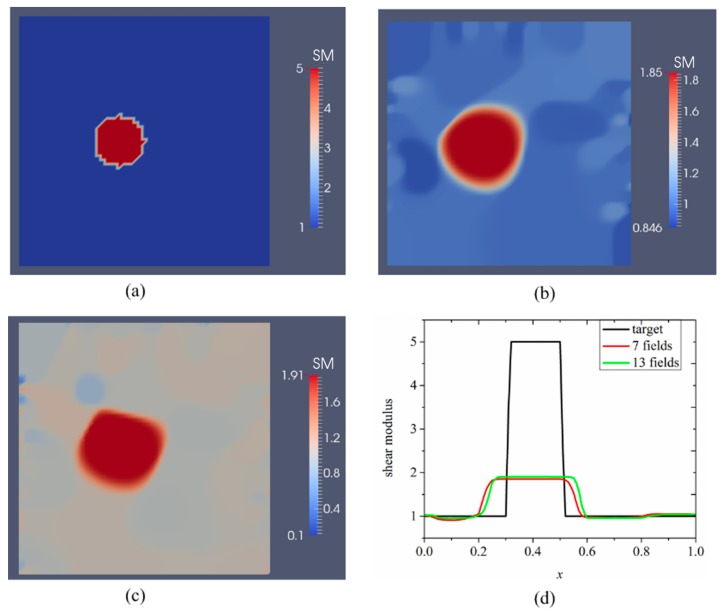
Shear modulus reconstructions for 1.0% noise. (**a**) target shear modulus distribution for comparison; (**b,c**) reconstructed shear modulus distribution using 7 and 13 boundary displacement data sets, respectively; (**d**) shear modulus plot over the horizontal line through the center of the inclusion for the target and reconstructed shear modulus distribution (unit in the scale bar: 10 kPa). Note: “SM” stands for shear modulus.

Figure 2 and Figure 3 show the reconstructed shear modulus distribution with respect to the noise levels of 0.1%, and 1%, respectively. In both figures, b, c represent the results for seven and 13 boundary displacement data sets, respectively. Seven displacement boundary data sets are obtained according to Figure 1a, three boundary displacement data sets are obtained according to Figure 1b, and three boundary displacement data sets are obtained according to Figure 1c.

The shear modulus values are plotted over the horizontal line passing through the center of the small inclusion in Figure 2d and Figure 3d. The regularization factors for Figure 2 and Figure 3 were chosen to be 10^−11^ and 10^−10^, respectively. In Figure 2, the reconstructions reveal that the location of the inclusion can be detected and the shape of the inclusion is well preserved. However, the inclusion seems to be larger than the target and the reconstructed shear modulus value of the inclusion is underestimated. Furthermore, with increasing number of displacement data sets, both the shape and the shear modulus value of the inclusion slightly improve, as shown in Figure 2b,c. More precisely, the reconstructed shear modulus value in the inclusion increases slightly from 2.07 to 2.17 and the recovered inclusion becomes more circular as shown in Figure 2c. We have also performed the reconstruction without noise and observed that the shear modulus distribution is very similar to the reconstructions in Figure 2 (not shown here).

In Figure 3b, we observe that with 1% noise level the recovered inclusion is larger than in the previous example with 0.1% noise level. Furthermore, the background has stronger oscillations due to the higher noise level. The reconstructions with 1% noise do not improve much with increasing number of displacement data sets used in Figure 3c. Nevertheless, we are able to detect the location of the inclusion center.

To better analyze the accuracy of the reconstructions in Figure 2 and Figure 3, we define a relative error to quantitatively evaluate the error between the recovered and target shear modulus distributions that is, $e=\sqrt{\sum_{i=1}^{N_{n}}{\left(\mu_{i}-\mu_{i}^{\circ }\right)}^{2}/\sum_{i=1}^{N_{n}}{\left(\mu_{i}^{\circ }\right)}^{2}}\times 100\%$, where $N_{n}$, $\mu_{i}$ and $\mu_{i}^{\circ }$ are the total number of nodes throughout the problem domain, nodal recovered shear modulus and nodal target shear modulus, receptively. The relative error for each case presented in Figure 2 and Figure 3 are shown in Table 1. Table 1 illustrates that increasing the number of boundary displacement datasets and decreasing the noise level improves the mapped shear modulus only slightly for case 1.

### 3.2. Case 2: A Semi-Circle Model with One or Two Inclusions

**Figure 4:**
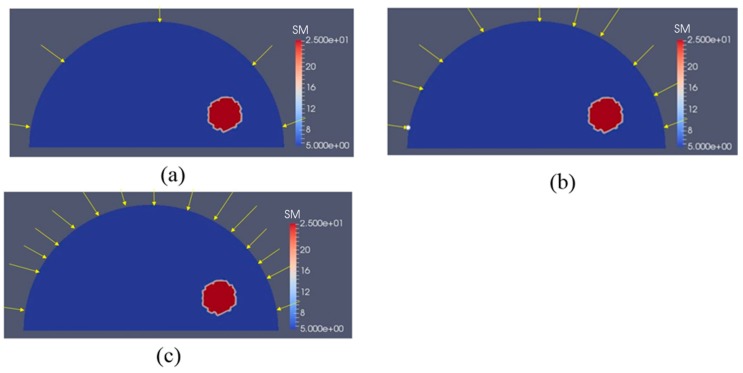
The problem domain for a semi-circle with a stiff inclusion surrounded by a soft background. The yellow arrows indicate the indentation locations, and measured boundary displacements are simulated on the top curve. (**a**) 5 arrows representing 5 sequentially applied forces to obtain boundary displacement data sets; (**b**) 10 arrows representing 10 sequentially applied forces to obtain boundary displacement data sets; and (**c**) 15 arrows representing 15 sequentially applied forces to obtain boundary displacement data sets (unit in the scale bar: kPa). Note: “SM” stands for shear modulus.

**Figure 5:**
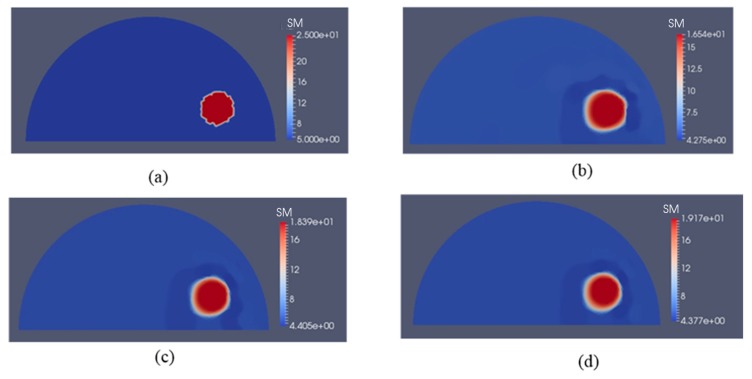
Shear modulus reconstructions without noise in boundary displacements. (**a**) target shear modulus distribution for comparison; (**b**–**d**) reconstructed shear modulus distribution using 5, 10 and 15 boundary displacement data sets, respectively (unit in the scale bar: kPa). Note: “SM” stands for shear modulus.

The second example in Figure 4 is a semi-circle with an inclusion that can be thought of as an idealized breast with an idealized tumor mimicking inclusion. The radii of the semi-circle and the inclusion are 7.5 cm and 1 cm, respectively. This problem domain is discretized with 7632 linear triangular elements. The exact shear moduli of the background and inclusion are 5 kPa and 25 kPa, respectively. To solve the forward problem in elasticity, we fix the bottom edge and apply indentations with a nodal force of 0.27 N on the top curved edge sequentially (the location and direction of each indentation are indicated by a yellow arrow in Figure 4a–c). Similar to the first case, the force will induce a small deformation of the simulated phantom. In this case, we assume that boundary displacements can be measured on the entire top curved edge. Figure 5b–d represent the recovered shear modulus distributions with 5, 10, and 15 boundary displacement data sets, respectively. In this case, no noise is introduced and the regularization factor is chosen to be 10^−11^. In general, we observe that the inclusion shape can be visualized well, while its shear modulus value is significantly underestimated by about 20%. Additionally, increasing the total number of displacement fields slightly improves both the reconstructed shear modulus value and the shape of the inclusions. The mapped shear modulus value in the inclusion increases from about 16.5 kPa to 19.1 kPa using 15 as shown in Figure 5d. It is also notable that the reconstructed shear modulus value in the inclusion reaches approximately 80% of the target value.

**Figure 6:**
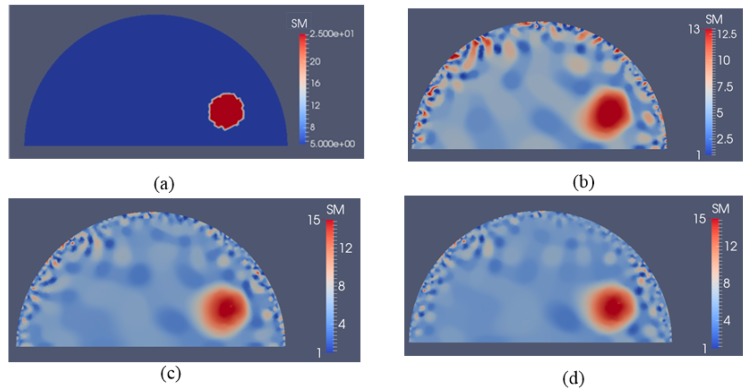
Shear modulus reconstructions with 1% noise. (**a**) target shear modulus distribution for comparison; (**b**–**d**) reconstructed shear modulus distribution using 5, 10, and 15 boundary displacement data sets, respectively (unit in the scale bar: kPa). Note: “SM” stands for shear modulus.

Figure 6b–d represent the recovered shear modulus distributions for a noise level of 1% with 5, 10, and 15 displacement data sets, respectively, for a regularization factor of 10^−10^. Compared to the case without noise, the recovered shear modulus distribution degrades significantly. The shear modulus value in the inclusion is roughly 15 kPa and does not change much with varying number of boundary displacement data sets. We also observe strong oscillations occurring throughout the problem domain, in particular close to the curved edge.

**Figure 7:**
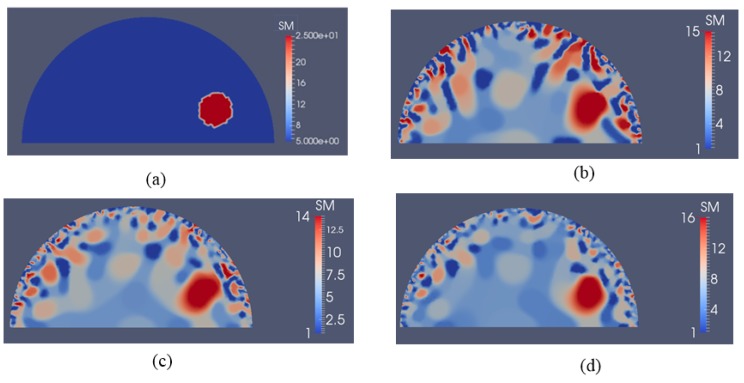
Shear modulus reconstructions with 5% noise. (**a**) target shear modulus distribution for comparison; (**b**–**d**) reconstructed shear modulus distribution using 5, 10, and 15 boundary displacement data sets, respectively (unit in the scale bar: kPa). Note: “SM” stands for shear modulus.

Figure 7b–d represent shear modulus reconstructions for a very high noise level of 5% with 5, 10 and 15 boundary displacement datasets, respectively. A regularization factor of 10^−9^ was selected in this case. In comparison with the reconstruction with 1% noise level in Figure 6, we observe that the noise artifacts are significantly amplified, with peaks closer to the boundary. We also computed the relative error for all cases presented in Figure 5, Figure 6 and Figure 7 as shown in Table 2. As expected, the accuracy in reconstruction results improves with a lower noise level as well as more displacement datasets.

**Figure 8:**
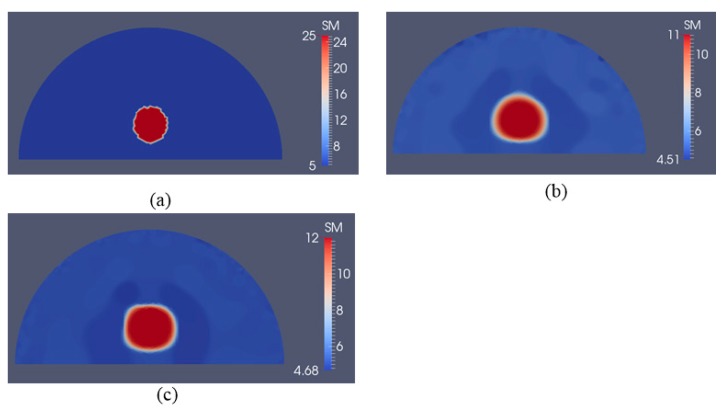
Shear modulus reconstructions with 0.1% noise. (**a**) target shear modulus distribution with varied inclusion depth in comparison to previous target problem domain in Figure 4a,b; (**b**,**c**) reconstructed shear modulus distribution using 5 and 10 boundary displacement data sets, respectively (unit in the scale bar: kPa). Note: “SM” stands for shear modulus.

**Figure 9:**
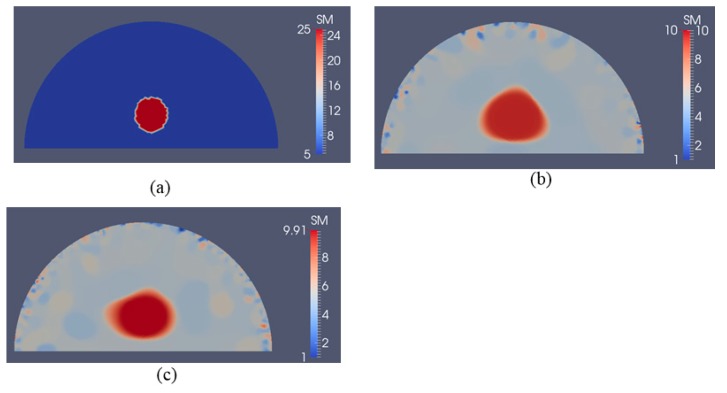
Shear modulus reconstructions with 1% noise. (**a**) target shear modulus distribution with varied inclusion depth in comparison to previous target problem domain in Figure 4a,b; (**b**,**c**) reconstructed shear modulus distribution using 5 and 10 boundary displacement data sets, respectively (unit in the scale bar: kPa). Note: “SM” stands for shear modulus.

In Figure 8a, we test a slightly different target problem domain from the previous one in Figure 4a,b, where the location of the stiff inclusion is positioned further away from the boundary. The boundary conditions, i.e., the applied force boundaries are the same as in the previous examples as well. The reconstructed shear modulus distribution is given in Figure 8b,c for 5 and 10 boundary displacement data sets, respectively, with a noise level of 0.1%. The regularization factor was chosen to be 10^−10^. We observe that the inclusion can be recovered despite its deeper location and being further away from the top boundary. In Figure 9, we increase the noise level to 1% using the same number of boundary displacement data sets, but increase the regularization factor to 5 × 10^−10^. The reconstructed shear modulus values deteriorate together with the shape of the inclusion compared to the previously lower noise level. Nevertheless, the inclusion shape and location are detectable.

**Figure 10:**
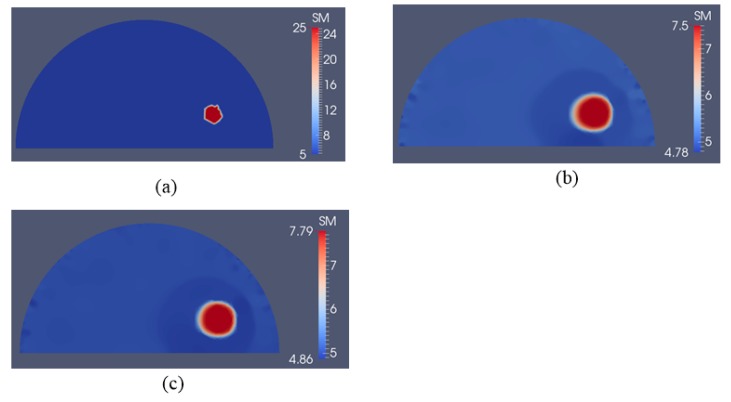
Shear modulus reconstruction with 0.1% noise. (**a**) target shear modulus distribution with a smaller inclusion radius of 0.5 cm is defined to study detectability of the inclusion to its size; (**b**,**c**) reconstructed shear modulus distribution using 5 and 10 boundary displacement data sets, respectively (unit in the scale bar: kPa). Note: “SM” stands for shear modulus.

**Figure 11:**
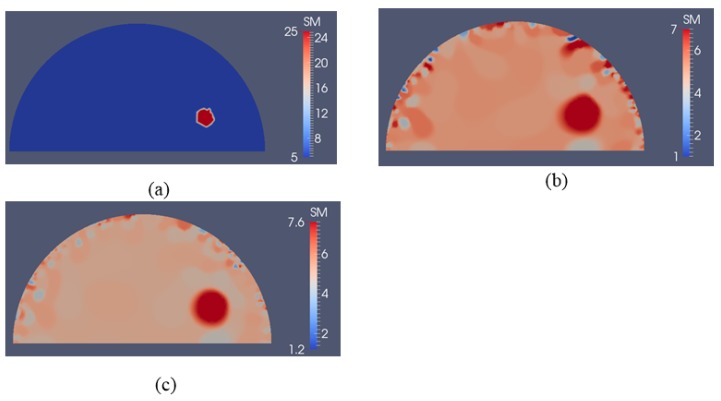
Shear modulus reconstruction with 1% noise. (**a**) target shear modulus distribution with a smaller inclusion radius of 0.5 cm is defined to study detectability of the inclusion to its size; (**b**,**c**) reconstructed shear modulus distribution using 5 and 10 boundary displacement data sets, respectively (unit in the scale bar: kPa). Note: “SM” stands for shear modulus.

To test the sensitivity to detect smaller inclusions, we have reduced the size of the inclusion to a radius of 0.5 cm in Figure 10a. The applied forces were the same as in Figure 4a,b. The reconstructed shear modulus distributions are shown in Figure 10b,c for 5 and 10 boundary displacement data sets, respectively, with a noise level of 0.1% and a regularization factor of 3 × 10^−10^. In Figure 11, we increase the noise level to 1% for the same displacement boundary data sets using a regularization factor of 7 × 10^−10^. Overall, we observe that the location and shape of the inclusion is preserved, while the size is overestimated and the shear modulus value in the inclusion is underestimated.

**Figure 12:**
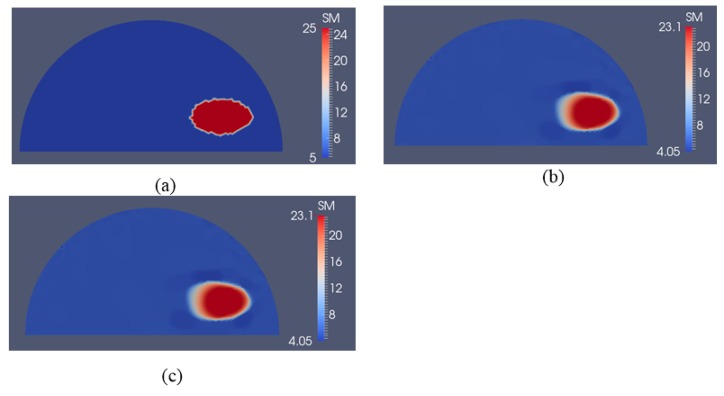
Shear modulus reconstructions with 0.1% noise. (**a**) target shear modulus distribution with an elliptic shaped inclusion is defined to study detectability of the inclusion shape; (**b**,**c**) reconstructed shear modulus distribution using 5 and 10 boundary displacement data sets, respectively (unit in the scale bar: kPa). Note: “SM” stands for shear modulus.

**Figure 13:**
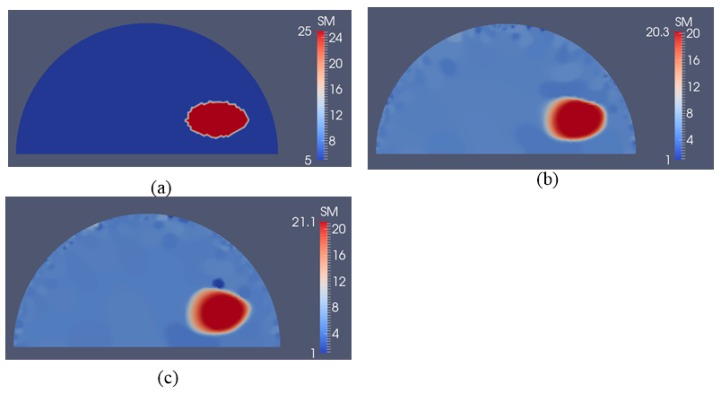
Shear modulus reconstructions with 1% noise. (**a**) target shear modulus distribution with an elliptic shaped inclusion is defined to study detectability of the inclusion shape; (**b**,**c**) reconstructed shear modulus distribution using 5 and 10 boundary displacement data sets, respectively (unit in the scale bar: kPa). Note: “SM” stands for shear modulus.

To test shape detectability of this approach, we define the target problem domain given in Figure 12a with an elliptic shaped inclusion. We apply the same boundary conditions as in Figure 4a,b and add 0.1% noise to boundary displacements. The reconstructed shear modulus distributions are shown in Figure 12b,c for 5 and 10 boundary displacement data sets, respectively, for a regularization factor of 5 × 10^−11^. We observe that the reconstructed inclusion shape follows the trend of an ellipse. In Figure 13, the noise level is increased to 1% and the regularization factor is chosen to be 5 × 10^−10^ and the shape deteriorates as anticipated, but an elliptic shape-like trend appears to be present.

**Figure 14:**
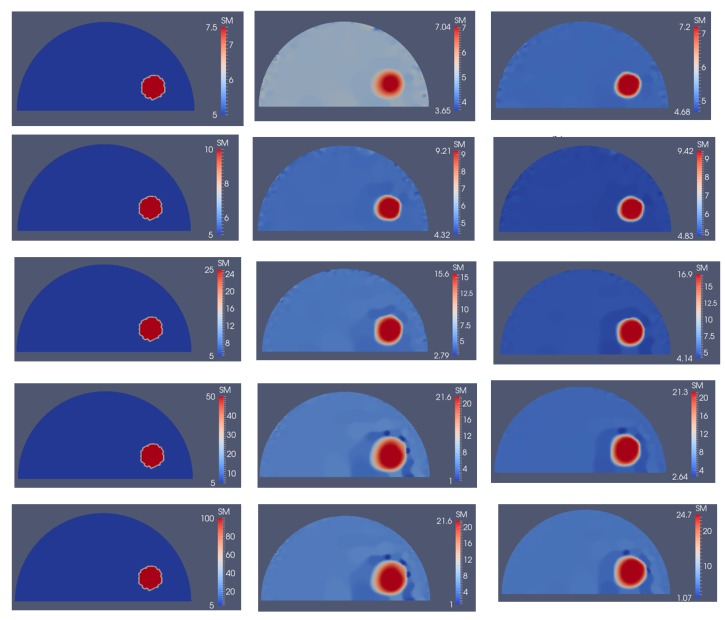
Problem domain with target shear modulus distribution is defined in the first column with varying shear modulus values in the inclusion from 7.5 kPa (top row) to 100 kPa (bottom row) to test the feasibility range of stiffness detection. Column 2 and column 3 represent the shear modulus reconstructions with 5 and 10 boundary displacement data sets, respectively, using 0.1% noise.

**Figure 15:**
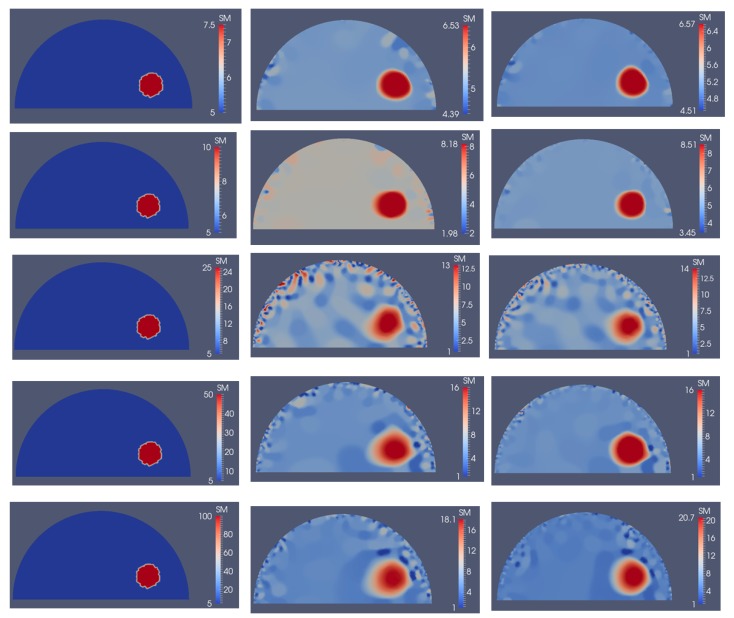
Problem domain with target shear modulus distribution is defined in the first column with varying shear modulus values in the inclusion from 7.5 kPa (top row) to 100 kPa (bottom row) to test the feasibility range of stiffness detection. Column 2 and column 3 represent the shear modulus reconstructions with 5 and 10 boundary displacement data sets, respectively, using 1% noise.

Next, we investigate the detectability of inclusions to varying stiffness contrasts. To this end, we specify target problem domains on the left column in Figure 14 with varying shear modulus values in the inclusion from 7.5 kPa to 100 kPa from the top to bottom row, respectively, while the background shear modulus value remains the same with 5 kPa. We utilize 5 and 10 boundary displacement data sets from solving the forward problem using force indentations according to Figure 4a,b and adding 0.1% noise. The reconstructions with 5 and 10 boundary displacement data sets are shown in columns 2 and 3, respectively. It appears that the stiffness contrast ratio of 2 according to row 2 in Figure 14 yields the best reconstructions. Decreasing or increasing the stiffness contrast ratio will compromise the accuracy of the shear modulus reconstructions. For the target shear modulus inclusion values of 50 and 100 (see last two rows in Figure 14), the reconstructed shear modulus values are very similar. The regularization factors were selected to be the same for each row in Figure 14 with 10^−10^, 10^−10^, 5 × 10^−11^, 5 × 10^−11^ and 5 × 10^−11^ starting from the top row down to the bottom row. Similarly, in Figure 15, the sensitivity of the reconstructions to the stiffness inclusion to the background ratio was analyzed for a noise level of 1%. The regularization factors from the top row to the bottom row were 5 × 10^−9^, 2 × 10^−9^, 1 × 10^−10^, 3 × 10^−10^ and 3 × 10^−10^, respectively. Increasing the noise level to 1% appears to yield the best reconstructions for a stiffness contrast of 1.5, shown in the first row of Figure 15.

**Figure 16:**
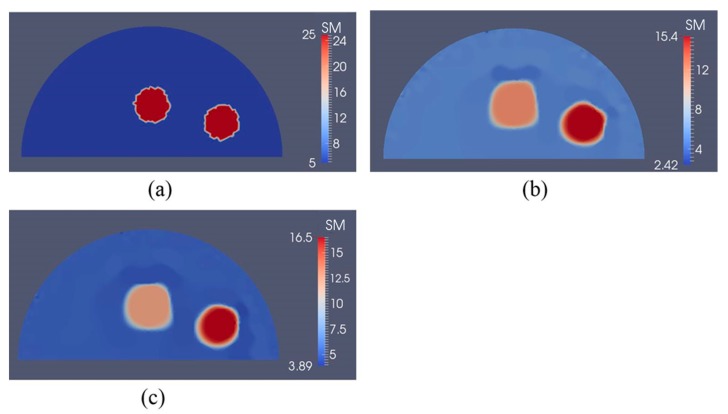
Shear modulus reconstructions with 0.1% noise. (**a**) target shear modulus distribution for comparison; (**b**,**c**) reconstructed shear modulus distribution using 5 and 10 boundary displacement data sets, respectively (unit in the scale bar: kPa). Note: “SM” stands for shear modulus.

**Figure 17:**
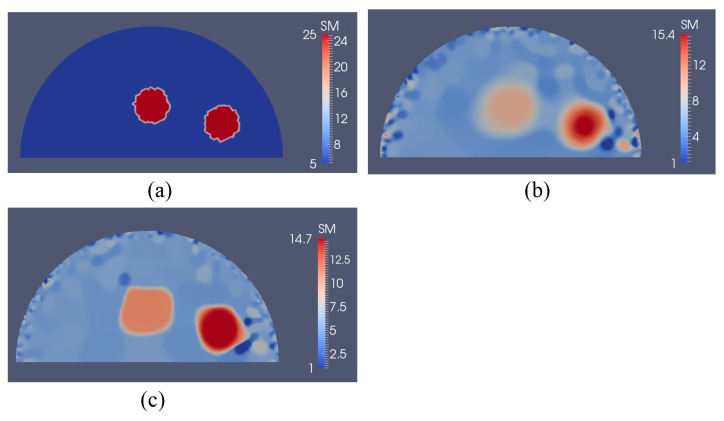
Shear modulus reconstructions with 1% noise. (**a**) target shear modulus distribution for comparison; (**b**,**c**) reconstructed shear modulus distribution using 5 and 10 boundary displacement data sets, respectively (unit in the scale bar: kPa). Note: “SM” stands for shear modulus.

**Figure 18:**
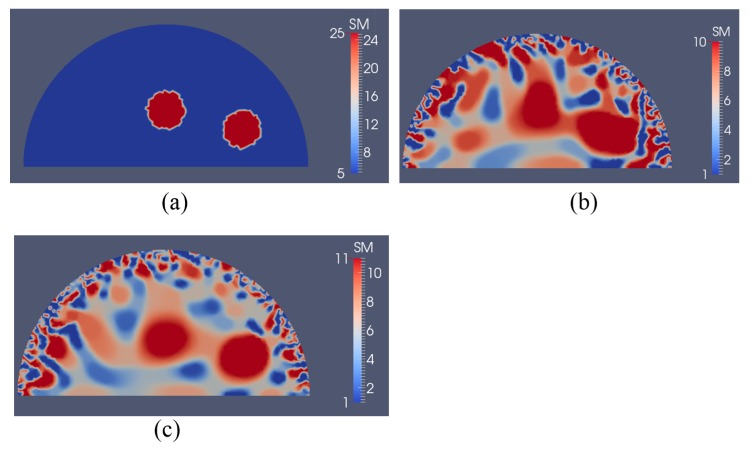
Shear modulus reconstructions with 5% noise. (**a**) Target shear modulus distribution for comparison; (**b**,**c**) reconstructed shear modulus distribution using 5 and 10 boundary displacement data sets, respectively (unit in the scale bar: kPa). Note: “SM” stands for shear modulus.

In Figure 16a, we have two stiff inclusions with shear modulus values of 25 kPa. In this case, we also apply radial indentations of 0.27 N and simulate displacement measurements on the top boundary edge. We utilize 5 and 10 displacement data sets in the presence of 0.1% noise to solve the inverse problem and the mapped shear modulus distributions are shown in Figure 16b,c, respectively. The regularization factor was chosen to be 10^−10^. The reconstructions reveal that both inclusions can be visualized and detected, while the shear modulus values are significantly underestimated. Furthermore, we note that the stiffness contrast of the left inclusion is more underestimated than that of the right inclusion. This is likely due to boundary sensitivity thoroughly discussed by the authors in [9,10,11]. In Figure 17 and Figure 18, we increase the noise level to 1% and 5%, respectively. The regularization factor for 1% noise level is chosen to be 3 × 10^−10^ and for 5% is chosen to be 5 × 10^−10^. While the shear modulus reconstruction with the high noise level of 5% is dominated by noise artifacts, the inclusions can be visualized to some extent. In Table 3, we compute the relative error for every case with two inclusions presented in Figure 16, Figure 17 and Figure 18 and we observe a similar trend that the accuracy in reconstruction results improves with a lower noise level as well as more displacement datasets observed in Table 2.

## 4. Discussion

In this work, a quantitative Mechanics Based Tomography (MBT) approach was introduced to characterize the shear modulus distribution using solely boundary displacements together with force information, and its feasibility has been tested using various simulated experiments. The inverse problem is posed as a minimization problem subject to the constraint of the equilibrium equations in elasticity. Unlike most inverse algorithms requiring measured displacements throughout the entire domain, i.e., full field displacements, the method presented in this paper merely requires measurements on the boundaries. This facilitates data collection for engineering materials by using digital cameras and a digital image correlation system, yielding a low cost imaging modality. In addition, displacements on the boundary can be conveniently measured with high resolution [25].

In our previous publications [22,23], we utilized displacement indentations as boundary conditions. Thus, the resulting shear modulus distribution was only recovered up to a multiplicative factor. In this paper, we assumed that the applied force is known, leading to quantitatively/absolutely reconstructed shear modulus values. One of the challenges we faced here was the sensitivity of the optimization method to the initial guess, while, for the relative shear modulus reconstructions, the optimization method converged for a wide range of initial guesses.

The first case could represent a tissue engineered material, where growth and remodeling of tissue scaffold by cells has progressed spatially. Thus, the inclusion could represent a hypothetical overproduction of collagen fibers, while the background could represent lower density of collagen fiber accumulation. This simulated case represents a challenging problem domain, since the inclusion is small and significantly away from the boundary edges (see Figure 1, Figure 2 and Figure 3). We note that we do not make any assumptions about any presence of inclusions for all examples in this paper, but assume that the shear modulus is unknown on the finite element mesh nodes. Furthermore, for case 1, we only measure one side for each indentation, which carries very little information pertaining to its interior shear modulus distribution. Nevertheless, the inverse scheme presented in this paper is capable of characterizing the non-homogenous shear modulus distribution well in the presence of noise levels (0.1%) that are inherent in actual measurements using digital image correlation systems. The reconstruction results reveal that the inverse algorithms are sufficiently robust to detect the location as well as the shape of the inclusion, while they fail to accurately reconstruct the target shear modulus value. For the case with 1% noise (see Figure 3b,c), the reconstructed inclusion becomes much larger and the shear modulus value in the inclusion is further underestimated. It is notable that the target inclusion area times the target inclusion value is preserved in that it is equal to the area of the reconstructed inclusion times the area of the reconstructed shear modulus value in the inclusion. This may be due to a lack of known boundary displacements (only used on partial boundaries) leading to uniqueness issues. Adding additional boundary displacement data sets does not significantly improve the reconstructions. Thus, to ensure an accurate and unique solution, displacements from the entire boundary should be used as discussed in the next case or deformations induced that could lead to a unique reconstruction.

For the second case, we have modeled a semi-circle with a shear modulus of 5 kPa according to measured fatty tissues and representing an idealized breast. The semi-circle consists of a stiff inclusion with a shear modulus value of 25 kPa, representing an idealized cancerous tumor (see Figure 4, Figure 5 and Figure 6). Here, the deformation of the curved top boundary edge is assumed to be measured and used to solve the inverse problem. Since the bottom edge is fixed in both directions, we actually have used the entire displacement information on the boundary to solve the inverse problem. We conclude that this leads to a much better reconstructed inclusion compared to the previous case.

To show that this novel approach is not confined to one inclusion only, we have also tested the inverse algorithms for simulated experiments with two inclusions as shown in Figure 16, Figure 17 and Figure 18. Clearly, the inverse algorithms are still capable of mapping the inclusion shapes, but underestimate the shear modulus values as in the one inclusion case. We observe that the left inclusion in Figure 16 is more underestimated than the right inclusion. The reason for this is that the solution of the inverse problem is sensitive to boundary conditions when regularizing the problem as discussed in [9,10,11].

In addition, we have added various noise levels into the boundary displacements to test the robustness of the novel inverse scheme herein. For experimentally relevant noise levels of about 0.1%, we observe that (1) the shape and size of the inclusion can be well recovered if the inclusion is medium sized; (2) the shear modulus value in the inclusion is underestimated; (3) the shape of the inclusion is preserved; (4) the size of the reconstructed inclusion is significantly overestimated for very small inclusions; (5) the stiffness contrast improves for a target stiffness contrast of about 1.5 to 2 and dramatically deteriorates for stiffness contrasts beyond 10. The shear modulus reconstructions deteriorate significantly for higher noise levels, tested in this paper at up to about 5%.

**Figure 19:**
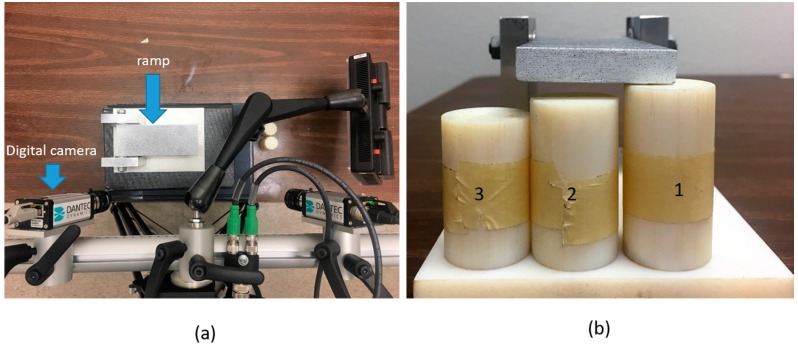
The experimental setup to perform noise analysis of the boundary displacement measurements utilizing a digital image correlation system. (**a**) top view of the experimental setup with digital cameras focusing on the ramp’s top face; (**b**) side view of the ramp with three columns having different height.

We also performed a simple experiment to estimate the noise level in boundary displacement measurements utilizing a digital image correlation system using digital cameras. In the experimental setup shown in Figure 19, the ramp is subject to rigid body rotation along the left end of the ramp. The height on the right end of the ramp was altered using the columns shown in Figure 19b. This will result in linear deflection of the top surface along the axial direction, used to validate the accuracy of the measurements obtained with the digital image correlation system. We defined a relative error $\sqrt{\sum_{i=1}^{T}{\left(z_{i}-z_{i}^{\circ }\right)}^{2}/\sum_{i=1}^{T}{\left(z_{i}^{\circ }\right)}^{2}}\times 100\%$ along the major axis of the ramp, where T, $z_{i}$ and $z_{i}^{\circ }$ are the total number of data points along the line, the measured deflection at those points and the curve fitting data from a linear function, respectively. We observe that the relative error is about 0.06% which is significantly lower than the noise levels significantly used in the simulations presented in this paper.

The inclusion to background stiffness contrast plays an important role in recovering the shear modulus distribution. We observe in Figure 14 and Figure 15 that the quality of the shear modulus reconstructions depends on the target stiffness ratio of inclusion to background. It is important to note that small stiffness contrasts of 7.5/5 (inclusion/background) can be well recovered. With increasing stiffness contrast ratio, the shear modulus reconstructions perform poorly; however, the shape of the inclusions is well-preserved in all cases for a noise level of 0.1%. Beyond a stiffness contrast ratio of 50 to 5, the shear modulus reconstructions do not differ much. This can be explained by the fact that the boundary displacements will not differ much either since the stiff inclusion behaves like a “rigid” object, i.e., the inclusion does not change its deformation field significantly beyond this stiffness ratio.

In all of the reconstructions presented in this paper, we added the same noise level for the displacement boundary, force and corresponding displacement indentation (i.e., displacement at force location), though they are not necessarily the same. However, from our experience, the reconstructions will not be sensitive to deviations in the noise level in force and corresponding displacement indentation. Furthermore, uncertainties in the location of force indentation are acknowledged and not investigated in this paper. Conducting the experiments carefully by marking the locations of force indentation, these uncertainties can be well controlled. To further elaborate on this, we pursue the following thought process: applying a force indentation at some predefined location will induce boundary displacements. Now, applying that same force indentation by some small incremental offset from the original location will result in a second set of boundary displacements. These two sets of boundary displacements will be very close, thus the resulting reconstructions would be anticipated to be close as well. As the offset of force location increases, the discrepancy between the boundary displacement sets will increase. This discrepancy can be understood as some kind of noise level in the boundary displacements as analyzed in this paper, and the reconstructions will depend on this discrepancy. We note, however, that this “noise” level from the discrepancy of boundary displacements is not random as utilized in this paper. A future analysis of this uncertainty will provide insight for experimental design.

In this work, we assumed that the simulated solids are in two-dimensional space and in the state of plane strain. Real world applications are in three-dimensional space and their reduction to plane strain may not always be feasible. Thus, future efforts will focus on extending this approach to three dimensions. Since many boundary displacements are needed, this is computationally intensive and may require further optimization of the in-house written program. However, collecting boundary displacement data in three-dimensional space using digital cameras is relatively convenient. Furthermore, recording digital camera images on boundary displacements of shapes that are more complex than a block or a hemisphere may be conveniently conducted.

## 5. Conclusions

In this paper, we have presented a novel and quantitative Mechanics Based Tomography (MBT) approach to determine the shear modulus distribution using boundary displacements together with applied force information. The feasibility of this approach has been tested with various simulated experiments. We observe that we can detect the location of the inclusion with various noise levels and preserve the shape of the inclusion well in the presence of 0.1% white Gaussian noise level in the boundary displacements. The results also illustrate that the shear modulus value is underestimated, and its inclusion size is larger than the target inclusion when incomplete displacement boundary information is utilized in the inverse problem. When complete boundary data is utilized as displayed in case 2, the overall solution to the inverse problem becomes more unique. In fact, we observe that for the first case with a square domain, knowing displacements on a small boundary region does not yield a unique solution, despite the low noise level and a large number of boundary displacement data sets. More studies are required to enforce uniqueness with limited boundary region measurements. We have also observed that, with an increasing number of displacement datasets utilized, the reconstruction results will improve at lower noise levels when boundary displacements are known everywhere, while no significant improvements are observed for higher noise levels and displacement measurements at partial boundaries. In summary, this novel approach has the potential to nondestructively and quantitatively map the heterogeneous elastic property distribution by utilizing displacements measured only on the specimen’s boundary together with the force indentation measurements.

## Figures and Tables

**Table 1:** Relative L2 error for each case presented in Figure 2 and Figure 3.

Noise Level	Relative L2 Error	
	7 Displacement Datasets	13 Displacement Datasets
0.1%	41.51%	40.39%
1%	43.89%	43.48%

**Table 2:** Relative L2 error for the cases presented in Figure 5, Figure 6 and Figure 7.

Noise Level	Relative L2 Error		
	5 Displacement Datasets	10 Displacement Datasets	15 Displacement Datasets
0%	28.68%	23.91%	22.52%
1%	45.40%	40.66%	38.30%
5%	69.26%	56.25%	50.78%

**Table 3:** Relative L2 error for the cases presented in Figure 16, Figure 17 and Figure 18.

Noise Level	L2 Relative Error	
	5 Displacement Datasets	10 Displacement Datasets
0.1%	42.12%	39.83%
1%	48.24%	45.92%
5%	68.01%	61.29%
