# Erratum: Mei, Y., et al. Mechanics Based Tomography: A Preliminary Feasibility Study. *Sensors* 2017, *17*, 1075

**DOI:** 10.3390/s18020384

**Published:** 2018-01-29

**Authors:** Yue Mei, Sicheng Wang, Xin Shen, Stephen Rabke, Sevan Goenezen

**Affiliations:** 1 Department of Mechanical Engineering, Texas A&M University, College Station, TX 77843, USA; meiyue1989@gmail.com (Y.M.); sx1992@tamu.edu (X.S.); pokeg16@tamu.edu (S.R.); 2 Department of Mathematics, Texas A&M University, College Station, TX 77843, USA; sichengwang0223@gmail.com

The authors wish to correct Figure 12 and Figure 14 in their paper published in *Sensors* [1], doi:10.3390/s17051075, http://www.mdpi.com/1424-8220/17/5/1075. 

The authors apologize for any inconvenience caused to readers by these changes. The manuscript will be updated, and the original will remain online on the article webpage with a reference to this Erratum.

## Figures and Tables

**Figure 12:**
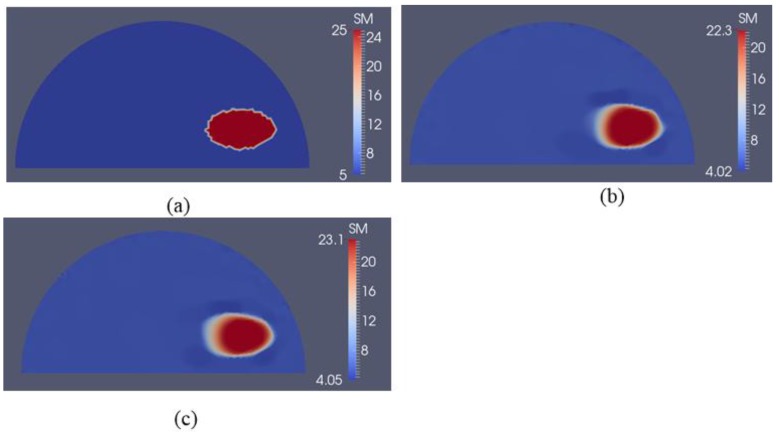
Shear modulus reconstructions with 0.1% noise. (**a**) Target shear modulus distribution with an elliptically shaped inclusion is defined to study detectability of the inclusion shape. (**b**,**c**) Reconstructed shear modulus distribution using 5 and 10 boundary displacement data sets, respectively (unit in the scale bar: kPa). Note: “SM” stands for shear modulus.

**Figure 14:**
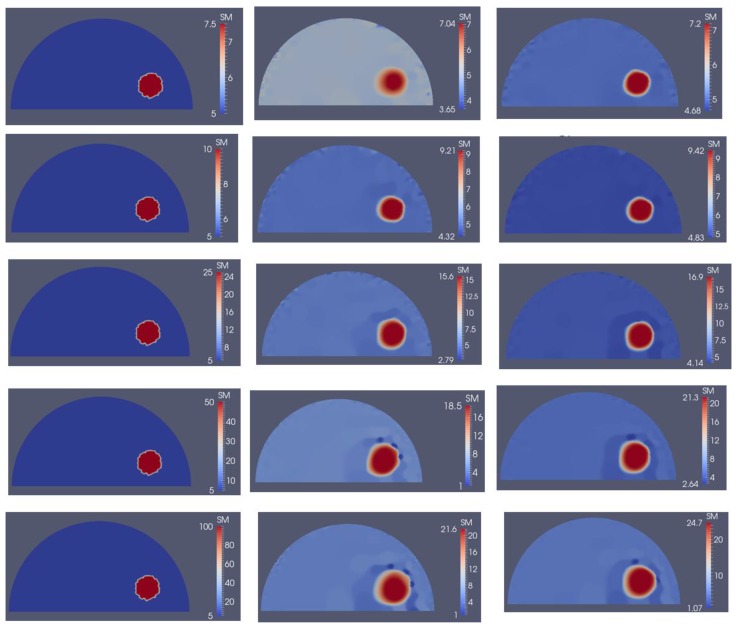
Problem domain with target shear modulus distribution is defined in the first column with varying shear modulus values in the inclusion from 7.5 (top row) to 100 kPa (bottom row) to test the feasibility range of stiffness detection. Columns 2 and 3 represent the shear modulus reconstructions with 5 and 10 boundary displacement data sets, respectively, using 0.1% noise.
